# The anatomy of sorrow: a spiritual, phenomenological, and neurological perspective

**DOI:** 10.1186/1747-5341-3-17

**Published:** 2008-06-17

**Authors:** Ronald Pies

**Affiliations:** 1 Department of Psychiatry, S.U.N.Y. Upstate Medical University, Syracuse, NY, USA; 2 Tufts University School of Medicine, Boston MA, USA

## Abstract

There is considerable controversy, both within and outside the field of psychiatry, regarding the boundaries of normal sadness and clinical depression. Furthermore, while there are frequent calls for a "pluralistic", comprehensive approach to understanding depression, few writers have tried to integrate insights from the spiritual, philosophical, and neurobiological literature. The author proposes that such a synthesis is possible, and that our understanding of ordinary sorrow and clinical depression is enriched by drawing from these disparate sources. In particular, a phenomenological analysis of sorrow and depression reveals two overlapping but distinct "lifeworlds". These differ in the *relational, temporal, dialectical*, and *intentional *realms. Recent brain imaging studies are also beginning to reveal the neurobiological correlates of sorrow and depression. As we come to understand the neurobiology of these states, we may be able to correlate specific alterations in "neurocircuitry" with their phenomenological expressions.

## Introduction

The field of psychiatry has always sought to incorporate insights from disciplines outside the realm of biology, notwithstanding the widespread notion that "biological psychiatry" is now the field's dominant paradigm. To be sure, recent advances in neurobiology–particularly in the area of mood disorders–have cast a bright light on the molecular and neurochemical bases of psychiatric illnesses.

To some degree, this has come at the expense of other modes of understanding. Indeed, some have upbraided modern-day psychiatry for ignoring the psychological, social and spiritual dimensions of emotional disorders. These attacks, in my view, distract us from the overriding task of integrating biological discoveries with a broader philosophy of emotional dysfunction. Insights from both the Western and Eastern spiritual traditions can help illuminate important aspects of ordinary sadness and pathological depression. A phenomenological analysis of these mood states can further enrich our understanding. Ultimately, I believe that a pluralistic view of mood disorders will aim at "mapping" experiential aspects of depression, such as hopelessness or self-deprecation, on to specific areas of brain dysfunction. In this paper, I try to provide a broad outline of such an integrated understanding of mood.

### A brief spiritual history of sorrow and depression

Psychiatrists and psychologists are hardly the only ones who have recognized the difference between clinical depression and "normal" sadness or sorrow. The distinction seems to be as old as recorded history. Surprisingly, in the Old Testament, the figure of King David presents us with portraits of *both *severe depression *and *normal bereavement. In Psalm 38, conventionally ascribed to David, the psalmist is lamenting his sins. He tells us that "There is no soundness in my flesh...no health in my bones because of my sin...my wounds grow foul and fester because of my foolishness, I am utterly bowed down and prostate; all the day I go about mourning...I groan because of the tumult of my heart." [1]. Modern diagnosticians would see in this description a picture quite consistent with an episode of major depression. In contrast, after the death of his beloved friend, Jonathan, the very same King David is far from "bowed down and prostrate". Rather, after a brief period of weeping and fasting, David is moved to write a passionately stirring dirge, known as "The Lament of the Bow" (2 Samuel 1:17–27), addressed to his lost friend: "How have the mighty fallen...I grieve for you, my brother Jonathan, you were most dear to me..." [1]. There is no trace, in David's lament, of the self-loathing and bodily decay found in Psalm 38. David's period of mourning after Jonathan's death represents roughly what modern-day mental health professionals would call "bereavement"–not clinical depression.

That life brings with it certain unavoidable or at least "expectable" sorrows is a concept found in Eastern religious thought, as well. In Buddhism, for example, we are told there are two roots of unhappiness in human existence: *dukha *and *tanha*. Dukha comprises the "...inevitable occasions of unhappiness" that come with human suffering, frailty, disease, loss of loved ones, and of course, death. Then there is *tanha*, which is translated as "blind demandingness": that part of our nature "...which leads us to ask of the universe...more than it is ready or even able to give." [2] Very roughly, we can see the precursors of normal and pathological sadness, respectively, in *dukha *and *tanha*.

Similarly, the 14^th ^century monk, Thomas ΰ Kempis (1380–1471) recognized that sorrow is sometimes appropriate. "Levity of heart and neglect of our faults," he wrote, "make us insensible to the proper sorrows of the soul." [3] Thomas asks, "Is there anyone who enjoys everything as he wishes? Neither you, nor I, nor anyone else on earth. There is no one in the world without trouble or anxiety, be he King or Pope."[3] Indeed, like many medieval theologians, Thomas saw this earthly existence as a vale of tears. He believed that, "...we often engage in empty laughter when we should rightly weep." [3]

Four centuries after Thomas ΰ Kempis, several Hassidic masters also distinguished between normal and abnormal degrees of sorrow. Rabbi Levi Yitzchak of Berditchev (1740–1810) wrote,

"There are two kinds of sorrow...When a man broods over the misfortunes that have come upon him... [and] cowers in a corner and despairs of help–that is a bad kind of sorrow..." In contrast, "...the other kind is the honest grief of a man who knows what he lacks." [4]

Similarly, writing at roughly the same time, Rabbi Simcha Bunam of Pshis'cha (1767–1827) recognized the distinction between "a broken heart" and what he termed "dejection":

"For it is a good thing to have a broken heart, and pleasing to God, as it is written: 'The sacrifices of God are a broken spirit...' [Psalm 51:19]...God does not entirely heal those who have broken hearts. He only eases their suffering, lest it torment and deject them. For dejection is not good and not pleasing to God. A broken heart prepares man for the service of God, but dejection corrodes service. *We must distinguish as carefully between the two as between joy and wantonness..." *[[4], p. 115, italics added].

Surprisingly, Rabbi Bunam seems to have foreseen not only our distinction between normal grief and clinical depression, but perhaps also that between *normal joy *and *hypomania *or *mania *("wantonness").

Of course, it is not always easy to tell "proper sorrows" from intense grief, "pathological" grief, or clinical depression. Indeed, it is very doubtful that these are strictly delineated categories. Furthermore, the nature of the putative "cause" or precipitating event is not a reliable predictor of where, on this emotional continuum, a given individual may end up. The loss of a loved one, for example, ordinarily provokes sorrow and a finite period of grief and mourning. Most mourners do not develop a severe, intractable clinical depression. Indeed, in the Judaic tradition, it is expected that after the seven days of mourning known as *shiva*, the bereaved will generally be ready to resume some "everyday" activities (while refraining, however, from any kind of celebration) [5].

There are, of course, many exceptions to the generally self-limited course of mourning; in principle, there are as many kinds of mourning as there are mourners. The great medieval philosopher, Moses ben Maimon (Maimonides, 1135–1204), appears to have developed a profound and prolonged depression, after the death of his beloved brother, David, in a shipwreck. Maimonides writes, in a letter dated from 1176,

"On the day I received that terrible news [of David's death], I fell ill and remained in bed for about a year, suffering from a sore boil, fever, and depression, and was almost given up. About eight years have since passed, but I am still mourning and unable to accept consolation...all joy has gone...whenever I see his handwriting or one of his letters, my heart turns upside down and my grief awakens again." [6]

### The phenomenology of sorrow

Just as the English scholar, Robert Burton, was able to develop an "anatomy of melancholy", we can develop a rough anatomy of sorrow. Burton himself recognized sorrow as related to, but *distinct from*, melancholy. Citing Hippocrates, Burton writes that sorrow is both "...mother and daughter of melancholy [depression]..." and that the two "tread in a ring...for sorrow is both cause and symptom of this disease" [7] In modern parlance, Burton describes a vicious circle, in which sorrow and depression are part of a mutually reinforcing feedback loop.

But though the boundaries between ordinary sorrow and significant depression are sometimes vague, there are experiential or "phenomenological" features that help us distinguish these conditions. For example, when we experience everyday sorrow, we generally feel–or at least are capable of feeling – *intimately connected with others*. Thus, Shakespeare has Romeo and Juliet parting in "...such sweet sorrow" (Act 2, scene 2). In contrast, when we experience severe depression, we typically feel outcast and alone. Sorrow, to use Martin Buber's terms, is an "I-Thou" or *relational *experience; clinical depression, a morbid preoccupation with "me". Indeed, William Styron, in *Darkness Visible*, describes depressed individuals as having "their minds turned agonizingly inward" [8]

The *sense of time *is also different in sorrow and depression. When we experience sorrow, we have the sense that, someday, it will end. As Psalm 30 tells us, "Weeping may last for the night, but joy returns in the morning." [1] Commenting on this passage, pastor John Phillips notes, "Sorrow is but a passing wayfarer who only tarries for a night; with the dawn, he leaves and joy takes his place." [9] In contrast, severe depression envelops us with the sense that it will last forever. Indeed, Dr. Nassir Ghaemi, drawing on the work of Leston Havens and Eugene Minkowski, has called attention to the sense of temporal distortion in depression; i.e., the subjective feeling that *time itself is slowed*. [10] Havens observes that for the depressed person, "...the future is lost, and the past becomes fixed, immovable, bad, the place of irredeemable mistakes." [11] Indeed, one recent empirical study demonstrated that during depression, the *experience of time itself is slowed*; in mania, it is accelerated. [12]

Sorrow, unlike severe depression, is curiously *ambivalent*: sorrow has the capacity to contain joy within it, or at least to find solace within its own essence. Sorrow, in this sense, is *dialectical: *it generates an inward "conversation" between hopeful possibility and foreclosure of hope. Thus, when Martin Luther was confronted with the imminent death of his beloved daughter, Magdalena, he is said to have uttered these words to the girl, as she lay in his arms:

"Lena dear, my little daughter, thou wilt rise again and shine like a star–yea, as the sun! I am happy in the spirit, but in the flesh I am very sorrowful." [13]

Another experiential difference between sorrow and depression is brought home in an anecdote concerning the writer James Joyce, and his daughter, Lucia, who was eventually diagnosed with schizophrenia. Although apparently apocryphal [14], the vignette makes an important existential distinction. Supposedly Joyce had brought Lucia to the eminent psychoanalyst, Dr. Carl Jung. Joyce was perplexed, regarding the difference between his own idiosyncratic thinking, and the convoluted thought processes of his daughter. Jung is said to have replied: "She falls. You *leap*."[14]

Indeed, we might say that depression is to sorrow as *falling *is to *leaping*. Put another way: we are *overtaken *by depression, but *give ourselves over *to sorrow. There is, in short, an *intentional *dimension to sorrow. The priest Francisco Fernández Carvajal tells us that, "...like love, sorrow is an act of the will, not a feeling." More than that, Fernandez insists, "Sorrow is a gift we must ask for." [15]

Sorrow, a gift? This counter-intuitive perspective is nicely elucidated by the psychotherapist and former Catholic monk Thomas Moore:

"Sorrow removes your attention from the active life and focuses it on the things that matter most. When you are going through a period of extreme loss or pain, you reflect on the people who mean the most to you instead of on personal success; and the deep design of your life, instead of distracting gadgets and entertainments. You may be more open to the beauty of your world as a relief from distress. Beauty is always present, but ordinarily you may not notice it because of your priorities or your absorption in other things." [16]

In contrast, it is very rare, though not unheard of, that severely depressed individuals consider their depression per se a "gift". Some, however, have found spiritual meaning or sources of creativity in their depression. Dr. Kay Redfield Jamison, a psychologist who suffers from bipolar disorder, has observed that

"Artistic expression can be the beneficiary of either visionary and ecstatic or, painful, frightening, and melancholic experiences. Even more important, however, it can derive great strength from the struggle to come to terms with such emotional extremes and from the attempt to derive from them some redemptive value." [17]

Nevertheless, the well-known association between bipolar disorder and creativity (16) does not mean that severe depression per se is a period of creativity. More typical of the depressive period and its effect on creativity is this description from essayist Virginia Heffernan:

"Depression brought to me a new rationing of resources: for every twenty-four hours, I got about three, then two, then *one *hour worth of life reserves–personality, conversation, motion." [18]

Psychiatrist Richard Berlin MD, editor of *Poets on Prozac: Mental Illness, Treatment, and the Creative Process*, has summarized his experience as follows:

"The idea that depression might enhance creativity is a myth, often based on the life stories and statements of deceased artists and writers... Contemporary poets who are alive and can tell us about their experience with depression are consistent in reporting that it was only after effective psychiatric treatment that they were able to create at their highest levels." (R.M. Berlin MD, personal communication, 1/27/08).

We have so far adumbrated four experiential dimensions that help differentiate clinical depression from sorrow; i.e., the *relational, temporal, dialectical*, and *intentional *realms. But this analysis is hardly exhaustive; indeed, we can hypothesize other phenomenological dimensions that may help distinguish clinical depression from ordinary sorrow. For example, do these states differ in the realm of *personhood*? As Berlin points out, severe depression interferes with *realistic self-assessment *[19]. For example, the severely depressed individual may describe herself as "a total nothing," "a complete failure," or "a big zero." In contrast, the sorrowful individual typically sees himself as a complex and full-fledged *person*; i.e., as one *bereft *but by no means rendered a *non-entity*.

Another (though perhaps related) phenomenological difference between sorrow and clinical depression may involve what I call *mortal vulnerability *– the sense, in severe depression, of being at the mercy of a hostile universe. This is wonderfully expressed in these lines from a poem entitled, "Depressive", by J.D. Smith:

Overshadowed by a blade of grass,

Soaked by one rain-drop,

Struck down by a dandelion seed.

Carried off by a sparrow...[20]

In summary, the *sorrowful *and the *severely depressed *inhabit two quite different phenomenological worlds, though the two "universes" intersect in certain experiential respects; for example, both the sorrowful and the depressed person will describe feelings of sadness and loss. The severely depressed person, however, endures a unique kind of suffering. Even though, as Paul Genova MD has observed, suffering may be beneficially "transformative" in *some *patients [21], others will simply be crushed by their suffering. Indeed, it is hard to find a better phenomenological description of such soul-killing suffering than in William Styron's account of his severe and intractable depression, in *Darkness Visible*:

"Death was now a daily presence, blowing over me in cold gusts. Mysteriously and in ways that are totally remote from normal experience, the gray drizzle of horror induced by depression takes on the quality of physical pain.... [the] despair, owing to some evil trick played upon the sick brain by the inhabiting psyche, comes to resemble the diabolical discomfort of being imprisoned in a fiercely overheated room. And because no breeze stirs this caldron, because there is no escape from the smothering confinement, it is entirely natural that the victim begins to think ceaselessly of oblivion... In depression the faith in deliverance, in ultimate restoration, is absent..." [8]

### A bridge from bereavement to clinical depression

A recent and very influential book, *The Loss of Sadness*, has argued that psychiatrists, over the last few decades, have "medicalized" sadness–in effect, lumping normal, adaptive sadness in with clinical depression, by failing to appreciate the emotional context in which depression takes place [22]. To be sure, the criteria for depressive disorders in DSM-IV are almost certainly too inclusive, and are undoubtedly in need of refinement. For example, current criteria conflate cases in which major depression has been present for only two weeks with those that have been present a year or more. The categorical approach of the DSM system also tends to create "pigeon holes" and procrustean checklists of symptoms.

Notwithstanding problems with the DSM system, psychiatrists have been in the forefront of distinguishing the subtle nuances of sorrow, grief, and depression, as well as calling upon us to appreciate the *experiential aspects *of the patient's mood states.

For example, Dr. Naomi Simon and her colleagues at Massachusetts General Hospital have described what they term "Complicated Grief" (CG)–sometimes referred to as "pathological" or "traumatic" grief [23]. The construct of traumatic grief dates from antiquity. Indeed, we find traumatic grief eloquently represented in Homer's *Iliad*, as psychiatrist Jonathan Shay has shown in his classic work, *Achilles in Vietnam *[24]. (Curiously, Horwitz and Wakefield see Achilles' grief as a prototype of "normal sadness" [22]. From the clinical standpoint, this hardly seems plausible). In the *Iliad*, we find this description of Achilles' grief, after the death of his beloved friend, Patroclus:

"A black cloud of grief swallowed up Achilles. With both hands he scooped up soot and dust and poured it on his head, covering his handsome face with dirt, covering his sweet-smelling tunic with black ash. He lay sprawling–his mighty warrior's massive body collapsed and stretched out in the dust. With his hands, he tugged at his own hair, disfiguring himself." [Book 18, lines 26–33] [25]

Things go downhill from there: Achilles essentially goes "berserk" and commits atrocities against his enemies, the Trojans. When Achilles says to his mother, Thetis, "Then let me die, since I could not prevent the death of my companion..." (Book 18, lines 122–23) [23], he is expressing a key element in unresolved grief, described millennia later by Freud, in his 1917 essay, *Mourning and Melancholia *[26]. Unlike ordinary mourning or bereavement, pathological mourning (what Freud terms "melancholia") involves *profound guilt and self-reproach*. To oversimplify Freud's thesis considerably, the survivor *blames himself*, on some unconscious level, for the death of the loved one.

Dr. Simon and her colleagues have delineated a syndrome that bears a close resemblance to the historical notion of traumatic or pathological grief [23]. Complicated grief (CG) is understood as a set of symptoms lasting at least six months after the loss of a loved one, and consisting of :

• A sense of disbelief regarding the death

• Persistent, intense longing, yearning, and preoccupation with the deceased

• Recurrent intrusive images of the dying person; and

• Avoidance of painful reminders of the death.

Notice that the first three features are essentially *phenomenological *criteria; that is, they reflect, and must be elicited from, the patient's *subjective experiential account*. (Arguably, the "avoidance of painful reminders" might be inferred from the bereaved person's *behavior*). Similarly, Simon et al also note that many individuals with CG often report "anger and bitterness related to the death", and "feel estranged from other close friends and relatives". They "cannot find satisfaction in ongoing life" [23]. While some of these features may remind us of Styron's severe major depression, *only a little over half of Dr. Simon's CG patients meet the full DSM-IV criteria for major depressive disorder*. Fully a quarter of all CG patients have *no DSM-IV diagnosis at all*. Nonetheless, Simon and her colleagues find that severe CG can result in significant social and vocational impairment. Complicated grief might be regarded, in our present state of knowledge, as a kind conceptual bridge between *ordinary bereavement *and full-blown *major depression*.

### The biology of sorrow?

If sorrow, bereavement, pathological grief, and major depression are distinguishable clinically and phenomenologically, we might hypothesize that they also differ *biologically*. This might be investigated from two perspectives. On the one hand, we might regard these mood states not as discrete categories, but as conditions along a *continuum *or *spectrum *of dysphoric mood and impaired function. Ghaemi, for example, posits a "unipolar depressive spectrum" that distinguishes *acute *from *chronic *major depression; and *single *from *recurrent *episodes of major depression [27]. (The terms "continuum" and "spectrum" are often used interchangeably in the depression literature. Technically, however, a *continuum *denotes a *progression of values *or elements varying by minute degrees, such as blood pressure readings (120/80, 121/81, 122/82, etc.). A *spectrum *denotes an ordered arrangement by a particular *characteristic*, such as a spectrum of visible color wavelengths. For our purposes, however, this distinction is not critical). Based on the spectrum-continuum model, we might hypothesize that mood states and disorders would yield *subtle gradations *of biological differentiae, rather than black-and-white distinctions.

On the other hand, we might posit a *categorical *separation of mood states. The now outmoded "reactive" (exogenous) versus "endogenous" distinction is one example of a categorical classification of depression (though, in theory, one could envision subtle gradations of "endogenicity"). If we posit such a categorical separation of mood states, we might also hypothesize *binary *or *dichotomous biological differentiae*; e.g., "abnormal" versus "not abnormal" laboratory values. Indeed, Taylor and Fink have proposed that *melancholia *is a biologically distinct subtype of depression, characterized most notably by abnormal elevations of serum cortisol [28].

While it is too early to decide which model – *spectrum-continuum *or *categorical *– more accurately corresponds to "reality", we continue to amass data on the neurobiology of dysphoric mood states.

For example, the "neurocircuitry" of *major depressive disorder *has been investigated by several groups [29]. Although a detailed review of this voluminous literature is beyond the scope of this paper, the most robust and reproducible finding using positron emission tomography (PET) is that of *decreased metabolic activity in the frontal lobes*. Conversely, a return to normal frontal lobe activity is associated with improvement in the patient's depression [29]. Many other brain regions, including various subcortical and limbic areas, also show abnormalities in some studies of major depression.

Unfortunately, the neurocircuitry of *normal and spontaneous *sadness, sorrow, or grief has received considerably less study. Preliminary data suggest both regional similarities and differences between normal sadness and clinical depression. However, since most studies have involved some method of "sadness induction" using visual images or verbal cues [30], inferences regarding *spontaneous*, "everyday" sadness or grief are probably premature.

One partial exception is an intriguing study by Najib et al [31], which assessed nine women whose "romantic relationship" had ended within the preceding 4 months. Subjects were scanned using functional magnetic resonance imaging (fMRI) while they alternated between recalling a sad, ruminative thought about their loved one (acute grief state) and a neutral thought about another person they knew an equally long time. Acute grief was associated with *increased activity *in *posterior *brain regions, including the cerebellum, posterior brainstem, and posterior temporoparietal and occipital brain regions. In contrast, more *anterior *regions, such as the orbitofrontal cortex, showed *decreased *activity. Strikingly, *the higher the subject's baseline level of grief, the greater the decrease in anterior brain activity*. Some of these findings overlap with PET studies of depression [29]. However, in the Najib et al study, *acute grief *was associated with *decreased activity in the amygdala*, whereas most studies of *depression *have found *hyperactivity *in the amygdala [31]. Replication of this last finding in spontaneous states of sadness or bereavement might point to different neurobiological substrates, compared with depression.

In this regard, it is interesting that–in the author's experience – severely depressed individuals who have recovered or achieved remission with antidepressant therapy consistently report the ability to experience ordinary sorrow or sadness. This might be interpreted as reflecting differing neurobiological substrates for major depression and ordinary sadness. On the other hand, some research finds that, in patients who meet DSM-IV criteria for both major depression *and *bereavement, response to an antidepressant [bupropion] produces a *concomitant reduction in both depressive symptoms and intensity of grief *[32]. This could be consistent with some degree of "biochemical overlap" between depression and bereavement, in those who meet criteria for both conditions. However, this does not rule out neurobiological differences between those with *depression alone *versus those with *bereavement alone*.

Further support for the biological separation of normal sadness and clinical depression comes from very recent research on *deep brain stimulation *(DBS) in extremely refractory cases of major depression. DBS entails the implantation of a tiny device called a "brain pacemaker", which sends electrical impulses to specific parts of the brain. DBS has been approved by the U.S. Food and Drug Administration for use in the treatment of Parkinson's disease and other movement disorders [33]. A small pilot study by Mayberg and colleagues [34] found that chronic deep brain stimulation (DBS) of the *subgenual cingulate *region (Brodmann area 25) resulted in "a striking and sustained remission of depression" in four of six patients with very resistant depression. All patients met DSM-IV criteria for major depression, and all had failed to respond to at least four treatments for depression (medication, psychotherapy, or electroconvulsive therapy). It is noteworthy that, in an attempt to control for placebo effects, the researchers performed a "blinded discontinuation" of the DBS in one patient who had experienced an early and robust response to treatment. After a period of about a month–and despite sustained euthymia (normal mood) on the Hamilton Depression Rating Scale–the patient began to exhibit a progressive decrease in energy, initiative, and concentration. When the correct stimulation frequency was restored (with the patient still "blinded" to the procedure), the patient's energy, initiative and concentration returned to pre-discontinuation levels within a week.

One post-study finding from this research group is of crucial importance to the "differentiation hypothesis"; i.e., the view that clinical depression and normal sadness have differing neurobiological underpinnings. Dr. Helen Mayberg, one of the lead investigators, reports that, after these severely depressed patients improve with DBS, they are *fully able to experience the normal range of emotions*, including ordinary sadness. Dr. Mayberg has found that in these recovered patients, "...there is no interference with feelings of normal sadness...". (H. Mayberg MD, personal communication, 4/24/08). It is also intriguing that Mayberg's patients describe their post-DBS emotional reactivity as quite different from the emotional "blunting" many of them experienced while taking conventional serotonergic antidepressants or atypical antipsychotics (Mayberg, personal communication, 4/24/08).

Also consistent with the differentiation hypothesis is a study by O'Connor et al [35] that examined autonomic function in subjects with either bereavement or depression. The bereaved subjects had all lost a close friend or family member, within the past two years, with an average period of about five months since the death. The researchers found that bereaved subjects showed significantly greater heart rate *than either depressed or normal subjects*. Not only did this study suggest a specific pattern of cardiovascular response in bereavement, it also raised questions regarding the so-called "broken heart" phenomenon–the observation that some grieving individuals may experience sudden cardiac death. If such a link were proved, it would certainly cast doubt on the much-touted notion [20] that bereavement is simply a "normal", "healthy" or evolutionarily adaptive response to loss.

Najib et al [31] opine that we are already able–albeit in a simplistic way–to "map" various experiential aspects of depression on to specific brain regions. For example, abnormalities in hypothalamic activity appear to correlate with alterations in sleep, appetite, and neuroendocrine dysfunction. Hyperactivity in the amygdala, they believe, "...maps onto co-morbid anxiety and misperception of danger signals." [31]. Are we so far, then, from being able to map the patient's sense of *existential isolation, hopelessness*, or *defeat *[31] onto specific, abnormally functioning brain regions? The answer may become more apparent in the next few decades of brain research. However, the diagnostic and therapeutic implications of such knowledge are far from clear. It seems to me that whatever the PET or fMRI findings may be in such a "brave new world", the psychiatrist's existential encounter with the patient [36-38] will remain critical in determining both diagnosis and treatment.

Dr. Helen Mayberg, considering the available neurobiological data, has opined that, "...we don't know how sadness/grief fits into the continuum of major depression... [however] the notion of normal circuits in a state of dysequilibrium is at least tenable and the imaging data supports it." (H. Mayberg MD, personal communication, 5/08/08). Moreover–notwithstanding the preliminary state of the evidence–the hypotheses developed in this paper allow us to generate a number of empirically testable predictions. For example, I would predict that among depressed individuals who experience *severe distortions in the relational, temporal, dialectical, and intentional realms*, we are likely to find (a) a higher frequency of *treatment-resistant depression*; and (b) a higher frequency of markedly *abnormal findings on fMRI and PET imaging*. If such predictions are borne out, this may have important treatment implications. For example, severe distortions in the phenomenological realm may someday point us toward especially effective neurobiological or psychosocial interventions. In the mean time, the hypotheses developed here might encourage researchers to develop semi-structured interviews or rating scales, aimed at quantifying pathology in the phenomenological realm.

## Conclusion

Though our mythic and literary heritage depicts ordinary grief and clinical depression as more or less discrete existential categories, it seems more likely that these conditions lie along a complex spectrum or continuum of dysphoric states. Moving from less to more severe, we may distinguish *normal sadness or sorrow; normal grief; complicated (pathological) grief*; and *major depression *as gradations along this continuum. Though this continuum may be characterized by very subtle gradations, both clinical and phenomenological features can help us distinguish normal sadness from severe, clinical depression. The syndrome of "complicated grief" (pathological mourning) may serve as a conceptual and phenomenological bridge between ordinary sorrow or grief, and major depression.

That said, both the *components *and *boundaries *of such a proposed continuum may be subject to debate. For example, should we exclude states of "normal sadness" and simply consider more incapacitating dysphoric states? And can we ever express, in objective terms, the subtle gradations and almost endless range of human emotional states? Certainly, the continuum proposed here should not be reified or made into a rigid instrument of classification; it is, at best, a heuristic tool in service of understanding the patient.

However we answer these questions, I believe that an understanding of the phenomenological "lifeworld" of the patient [36-38] must be incorporated into pluralistic models of depression. In time, we may come to understand how the phenomenology of depression and "proper sorrows" relates to their neurobiological substrates. Indeed, I believe that a full understanding of sorrow and depression will synthesize insights from spiritual, phenomenological and neurobiological perspectives.

## About the author

Ronald Pies MD is Professor of Psychiatry and Lecturer on Bioethics and Humanities at S.U.N.Y. Upstate Medical University in Syracuse, New York, and Clinical Professor of Psychiatry at Tufts University School of Medicine in Boston, Massachusetts. He is the author of *The Ethics of the Sages *(Rowman & Littlefield) and *Everything Has Two Handles: The Stoic's Guide to the Art of Living *(Hamilton Books) as well as several textbooks on psychopharmacology. He is interested in the connection between mental health care and various spiritual traditions.

## Competing interests

The author declares that he has no competing interests.

## Authors' contributions

RP wrote the manuscript in its entirety
